# Supplementary material for: Dynalign II: common secondary structure prediction for RNA homologs with domain insertions
Source: Nucleic Acids Res. 2014 Nov 21;42(22):13939–48. doi: 10.1093/nar/gku1172 (PMC4267632; doi:10.1093/nar/gku1172)
Supplement: SUPPLEMENTARY DATA [file supp_gku1172_nar-02021-z-2014-File012.zip › manual/javadoc/RNAstructure_java_drawing/source/proxy/DotPlotBackend.html]

DotPlotBackend


JavaScript is disabled on your browser.


- Overview
- Package
- Class
- Tree
- Index
- Help

- Prev Class
- Next Class

- Frames
- No Frames

- All Classes

- Summary:
- Nested |
- Field |
- Constr |
- Method

- Detail:
- Field |
- Constr |
- Method


RNAstructure\_java\_drawing.source.proxy

## Class DotPlotBackend

- java.lang.Object
- - RNAstructure\_java\_drawing.source.proxy.DotPlotBackend

- ---

    

  ```
  public class DotPlotBackend
  extends Object
  ```

- - ### Field Summary

    Fields

    | Modifier and Type | Field and Description |
    | --- | --- |
    | `protected boolean` | `swigCMemOwn` |
    | `private long` | `swigCPtr` |
  - ### Constructor Summary

    Constructors

    | Modifier | Constructor and Description |
    | --- | --- |
    |  | `DotPlotBackend()` |
    | `protected` | `DotPlotBackend(long cPtr, boolean cMemoryOwn)` |
  - ### Method Summary

    Methods

    | Modifier and Type | Method and Description |
    | --- | --- |
    | `void` | `delete()` |
    | `protected void` | `finalize()` |
    | `String` | `getBounds()` |
    | `String` | `getColors()` |
    | `protected static long` | `getCPtr(DotPlotBackend obj)` |
    | `double` | `getCurrentMax()` |
    | `double` | `getCurrentMin()` |
    | `double` | `getDefaultMax()` |
    | `double` | `getDefaultMin()` |
    | `String` | `getDotData(int i, int j)` |
    | `String` | `getDotDataByLocation(int x, int y, double scale)` |
    | `String` | `getGridLine(int number)` |
    | `String` | `getLegendData()` |
    | `int` | `getLength()` |
    | `boolean` | `readData(String file, int strand)` |
    | `void` | `setColors(int colors)` |
    | `void` | `setRange(double minimum, double maximum)` |
    | `void` | `writePostscriptFile(String outFile)` |
    | `String` | `writeStructuresFile(String file, String outFile)` |
    | `void` | `writeSVGFile(String outFile)` |
    | `void` | `writeTextFile(String file)` |

    - ### Methods inherited from class java.lang.Object

      `clone, equals, getClass, hashCode, notify, notifyAll, toString, wait, wait, wait`

- - ### Field Detail


    - #### swigCMemOwn

      ```
      protected boolean swigCMemOwn
      ```


    - #### swigCPtr

      ```
      private long swigCPtr
      ```
  - ### Constructor Detail


    - #### DotPlotBackend

      ```
      public DotPlotBackend()
      ```


    - #### DotPlotBackend

      ```
      protected DotPlotBackend(long cPtr,
                    boolean cMemoryOwn)
      ```
  - ### Method Detail


    - #### delete

      ```
      public void delete()
      ```


    - #### finalize

      ```
      protected void finalize()
      ```

      **Overrides:**
      :   `finalize` in class `Object`


    - #### getBounds

      ```
      public String getBounds()
      ```


    - #### getColors

      ```
      public String getColors()
      ```


    - #### getCPtr

      ```
      protected static long getCPtr(DotPlotBackend obj)
      ```


    - #### getCurrentMax

      ```
      public double getCurrentMax()
      ```


    - #### getCurrentMin

      ```
      public double getCurrentMin()
      ```


    - #### getDefaultMax

      ```
      public double getDefaultMax()
      ```


    - #### getDefaultMin

      ```
      public double getDefaultMin()
      ```


    - #### getDotData

      ```
      public String getDotData(int i,
                      int j)
      ```


    - #### getDotDataByLocation

      ```
      public String getDotDataByLocation(int x,
                                int y,
                                double scale)
      ```


    - #### getGridLine

      ```
      public String getGridLine(int number)
      ```


    - #### getLegendData

      ```
      public String getLegendData()
      ```


    - #### getLength

      ```
      public int getLength()
      ```


    - #### readData

      ```
      public boolean readData(String file,
                     int strand)
      ```


    - #### setColors

      ```
      public void setColors(int colors)
      ```


    - #### setRange

      ```
      public void setRange(double minimum,
                  double maximum)
      ```


    - #### writePostscriptFile

      ```
      public void writePostscriptFile(String outFile)
      ```


    - #### writeStructuresFile

      ```
      public String writeStructuresFile(String file,
                               String outFile)
      ```


    - #### writeSVGFile

      ```
      public void writeSVGFile(String outFile)
      ```


    - #### writeTextFile

      ```
      public void writeTextFile(String file)
      ```


- Overview
- Package
- Class
- Tree
- Index
- Help

- Prev Class
- Next Class

- Frames
- No Frames

- All Classes

- Summary:
- Nested |
- Field |
- Constr |
- Method

- Detail:
- Field |
- Constr |
- Method
